# Supplementary material for: Treatment Costs of Colorectal Cancer by Sex and Age: Population-Based Study on Health Insurance Data from Germany
Source: Cancers (Basel). 2022 Aug 8;14(15):3836. doi: 10.3390/cancers14153836 (PMC9367511; doi:10.3390/cancers14153836)
Supplement: Supplementary file 1 [file cancers-14-03836-s001.zip › cancers-1694602-supplementary.pdf]

Supplementary Materials

Treatment Costs of Colorectal Cancer by Sex and Age: Population-Based Study on Health Insurance Data from Germany

Thomas Heisser, Andreas Simon, Jana Hapfelmeier, Michael Hoffmeister and Hermann Brenner

Supplementary Table S1. Definition of costs positions.

Setting	Definition
Inpatient	All inpatient costs charged for the complete hospital stay when CRC has been coded as primary or secondary diagnosis. Including all cost positions which are not considered CRC-related, e.g. dialysis, but have been conducted during the hospital stay.
Ambulatory	All outpatient costs charged for the complete outpatient case when CRC has been coded with a secure ICD-10 diagnosis. Including all cost positions which are not considered CRC-related but have been conducted during the time period of the whole outpatient case.
Medication	In medication setting, package units are multiplied with the price per unit. All ATC-codes from the following groups:<br>- Chemotherapies and cytostatic antibiotics: 'L01BC06','L01BC02','L01BC59','L01XA03','L01XX19','V03AF03','L01DC03'<br>- Innovative medicines (targeted therapies and monoclonal antibodies) L01XE21','L01XE15','L01XE07','L01XX44','S01LA05'; L01XC07', 'L01XC06','L01XC08','L01XC21','L01XC17','L01XC11','L01XC18', 'L01XC03'<br>- Other medication, possible CRC-related, defined as the remainder excluding the following ATC codes:<br>'D', 'G', 'H', 'P', 'R', 'S', 'M','A01', 'A02', 'A08', 'A10', 'A13', 'A14','B01', 'B02', 'B03', 'B04', 'B05', 'B06','C01', 'C02', 'C03', 'C04', 'C05', 'C06', 'C07', 'C08', 'C09','J04', 'J05', 'L03', 'L04', 'N03', 'N04', 'N07', 'V01', 'V03', 'V60''N05A', 'N06B', 'N06C', 'N06D', 'V09A', 'V09E', 'V09F', 'V09G', 'V09H'

Supplementary Table S2. Colorectal cancer cost distributions in colorectal cancer patients after diagnosis, stratified by sex and age.

Year after Diagnosis	No of Patients	Average Costs	25% Quantile	Median Costs	75% Quantile	No of Patients	Average Costs	25% Quantile	Median Costs	75% Quantile
	Men					women				
	**<70 age**					**<70 age**				
1	486	16,450	409	11,409	23,974	411	10,071	279	2529	15,829
2	336	3147	67	476	1081	283	1900	17	204	611
3	119	1809	88	363	797	91	2226	53	257	596
4	79	1203	34	198	439	55	765	14	89	364
	**≥70 age**					**≥70 age**				
1	360	16,375	7501	11,896	23,143	491	13,250	4549	11,230	18,015
2	285	1998	202	478	945	371	2009	201	498	961
3	120	1920	101	511	865	173	2122	182	437	899
4	94	1195	57	201	474	131	841	97	294	574

Supplementary Table S3. Colorectal cancer cost distributions in colorectal cancer patients prior to death, stratified by sex and age.

Year after Diagnosis	No of Patients	Average Costs	25% Quantile	Median Costs	75% Quantile	No of Patients	Average Costs	25% Quantile	Median Costs	75% Quantile
	Men					women				
	**<70 age**					**<70 age**				
1	165	34,351	7250	33,855	53,729	90	31,417	13,745	31,546	48,288
2	117	30,609	6615	25,369	46,902	56	23,041	1954	19,942	36,977
3	70	20,193	444	14,348	29,978	25	23,151	869	17,858	45,349
4	64	12,892	262	3346	16,976	23	10,759	300	2382	16,706
5	35	6696	131	598	8704	15	5835	82	1382	6979
	**≥70 age**					**≥70 age**				
1	393	14,463	565	2754	21,907	469	9930	677	2533	11,254
2	303	10,109	325	885	12,210	345	7180	337	1100	8244
3	191	4907	215	543	2324	215	3692	186	553	2358
4	176	5874	100	526	5180	199	3148	156	447	1719
5	108	3070	43	247	1066	121	2038	109	307	953

Supplementary Table S4. Total inpatient, outpatient and medication costs in colorectal cancer patients 1–5 years after diagnosis, stratified by sex and age (Total healthcare costs).

Year after Diagnosis	No of Patients	Median Age (years)	Average Costs in EUR Hospital	Ambulatory	Medication	Total
			**Men**			
**<70 age**						
1	486	59	15,025	1733	3487	20,245
2	336	60	3865	850	1720	6435
3	119	61	1906	779	1194	3879
4	79	62	707	526	1141	2374
**≥70 age**						
1	360	75	16,540	1818	2852	21,210
2	285	76	3458	1050	1732	6240
3	120	77	3278	1088	1466	5832
4	94	78	1502	712	1236	3450
			**Women**			
**<70 age**						
1	411	58	9172	1267	2670	13,109
2	283	59	1608	838	1493	3938
3	91	59	2041	946	1843	4831
4	55	62	1145	559	507	2211
**≥70 age**						
1	491	78	14,087	1519	2122	17,728
2	371	78	3557	1098	1737	6392
3	173	78	2387	1123	1967	5477
4	131	79	1058	635	881	2574

Costs as actually incurring in the period 2012–2016.

Supplementary Table S5. Total inpatient, outpatient and medication costs in colorectal cancer patients 1–5 years prior to death, stratified by sex and age (Total healthcare costs).

Year before Death	No of Patients	Median Age (years)	Average Costs in EUR			
			Hospital	Ambulatory	Medication	Total
			**Men**			
**<70 age**						
5	35	62	8441	358	3466	12,265
4	64	61	8482	906	7178	16,566
3	70	61	8470	1491	16,843	26,804
2	117	60	15,930	2203	19,962	38,094
1	165	60	23,189	2309	19,963	45,460
**≥70 age**						
5	108	81	4826	319	1507	6652
4	176	80	7616	495	2064	10,174
3	191	81	6068	827	3668	10,563
2	303	81	8200	1238	6024	15,462
1	393	81	16,854	1601	6932	25,387
			**Women**			
**<70 age**						
5	15	64	3886	729	4458	9073
4	23	62	9034	696	5310	15,040
3	25	63	10,221	1208	14,997	26,426
2	56	62	11,820	1886	16,462	30,168
1	90	60	25,100	2210	18,002	45,312
**≥70 age**						
5	121	82	3102	290	1472	4864
4	199	83	4412	490	1594	6496
3	215	84	4471	685	2424	7579
2	345	84	6648	1141	4030	11,819
1	469	84	13,458	1443	4358	19,259

Costs as actually incurring in the period 2012–2016.

Supplementary Table S6. Total inpatient, outpatient and medication costs in colorectal cancer patients prior to death, stratified by sex and age, excluding individuals with poor prognosis (diagnosed and died within 2012–2016).

Year before Death	No of Patients	Median Age (years)	Average CRC-related Costs in EUR				Average Total Healthcare Costs in EUR
			Hospital	Ambulatory	Medication	Total	
				Men			
**<70 age**							
5	35	62	4007	152	2538	6696	12,265
4	55	62	5198	388	5195	10,781	14,860
3	58	63	4701	834	11,272	16,807	23,551
2	66	63	8748	1453	18,571	28,772	36,028
1	83	62	12,140	1470	17,284	30,894	44,302
**≥70 age**							
5	108	81	2182	109	779	3070	6652
4	166	81	4001	224	1334	5560	9959
3	177	81	1317	398	2449	4164	9603
2	230	81	2400	587	4010	6997	12,590
1	252	82	4107	886	4456	9449	21,505
				Women			
**<70 age**							
5	15	65	2668	187	2980	5835	9073
4	22	64	5878	455	4513	10,846	15,292
3	22	66	9043	827	11,842	21,712	25,090
2	37	64	6162	990	13,557	20,710	28,603
1	44	64	7631	1698	16,374	25,703	43,478
**≥70 age**							
5	121	82	1514	109	415	2038	4864
4	188	83	2018	215	781	3014	6410
3	201	84	1646	334	1529	3508	7490
2	257	85	2038	533	2464	5034	9569
1	293	85	3928	722	2374	7024	16,544

Costs as actually incurring in the period 2012–2016.

Supplementary Table S7. Total inpatient, outpatient and medication costs in colorectal cancer patients prior to death, stratified by sex and age, only individuals with poor prognosis (diagnosed and died within 2012–2016) *.

Year before Death	No of Patients	Median Age (years)	Average CRC-related Costs in EUR				Average Total Healthcare Costs in EUR
			Hospital	Ambulatory	Medication	Total	
			**Men**				
**<70 age**							
4	5	63	10396	1739	15472	27607	30724
3	6	61	2992	3429	26797	33218	41054
2	28	61	19939	1992	10751	32682	41071
1	42	61	16635	2036	14042	32713	42230
**≥70 age**							
4	4	80	7842	997	2141	10981	13405
3	7	75	14439	1532	9723	25694	36614
2	45	78	13319	1540	6187	21045	26925
1	87	79	16508	1492	7350	25349	33609
			**Women**				
**<70 age**							
2	11	60	9379	2308	17996	29683	35972
1	23	61	22119	1871	15559	39549	49506
**≥70 age**							
4	7	82	4915	182	439	5536	7548
3	10	85	7208	835	673	8715	11177
2	59	83	8191	1143	5601	14935	19319
1	123	83	9626	1298	3939	14863	22235

* no data available for women <70 years 3–4 years prior to death

Costs as actually incurring in the period 2012–2016.

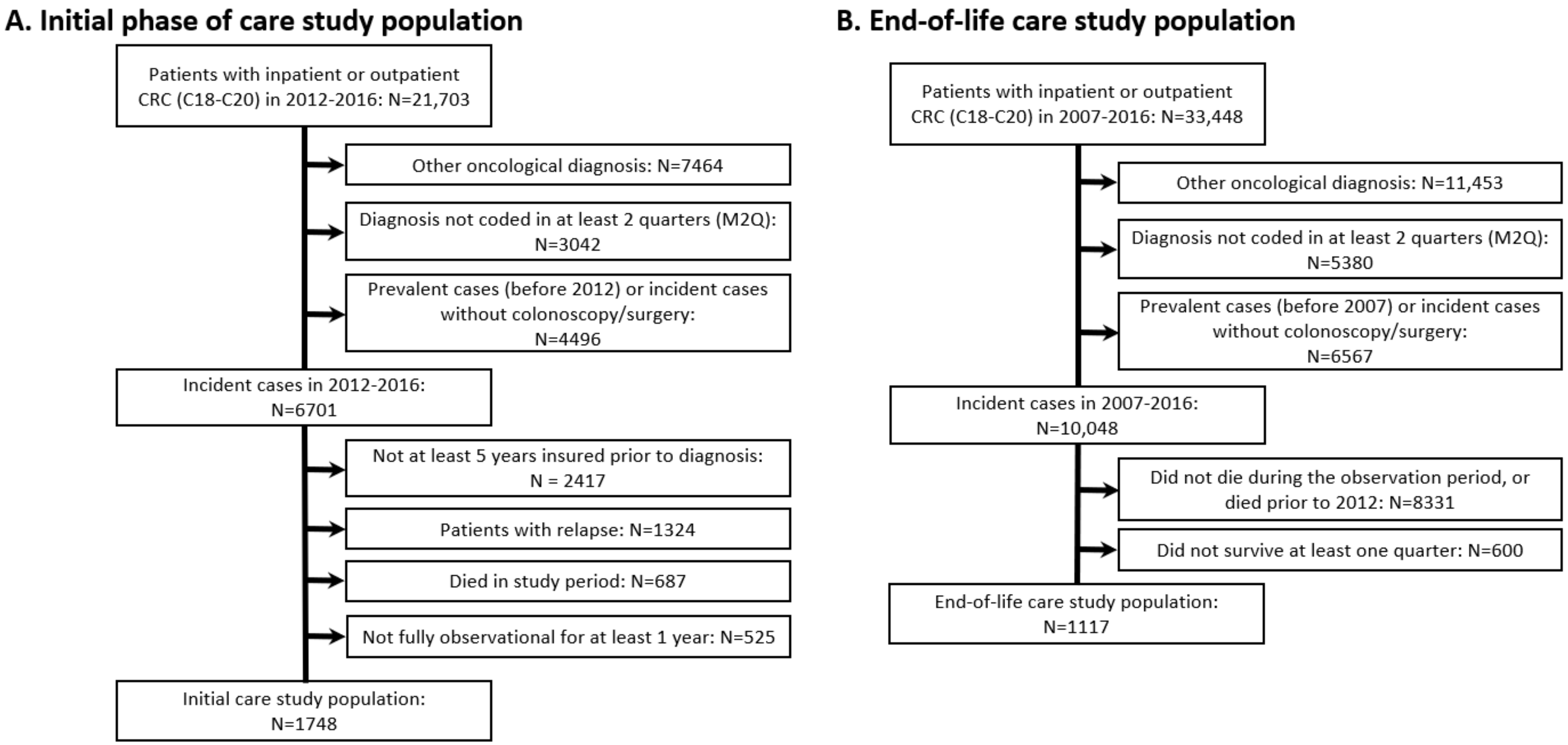

CRC, colorectal cancer

Supplementary Figure S1. Patient flowcharts.

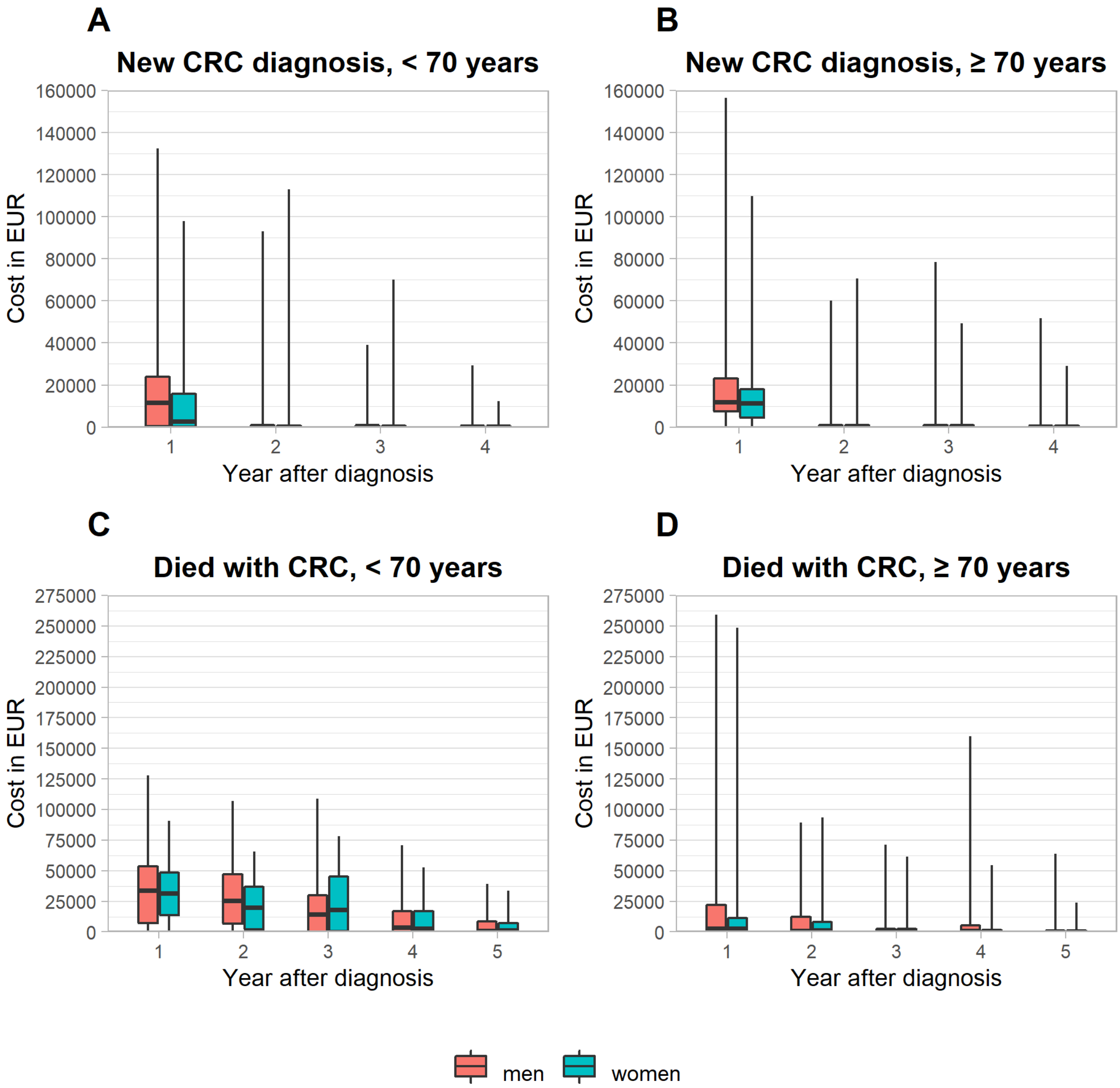

CRC, colorectal cancer

Supplementary Figure S2. Boxplots of total colorectal cancer cost distributions.

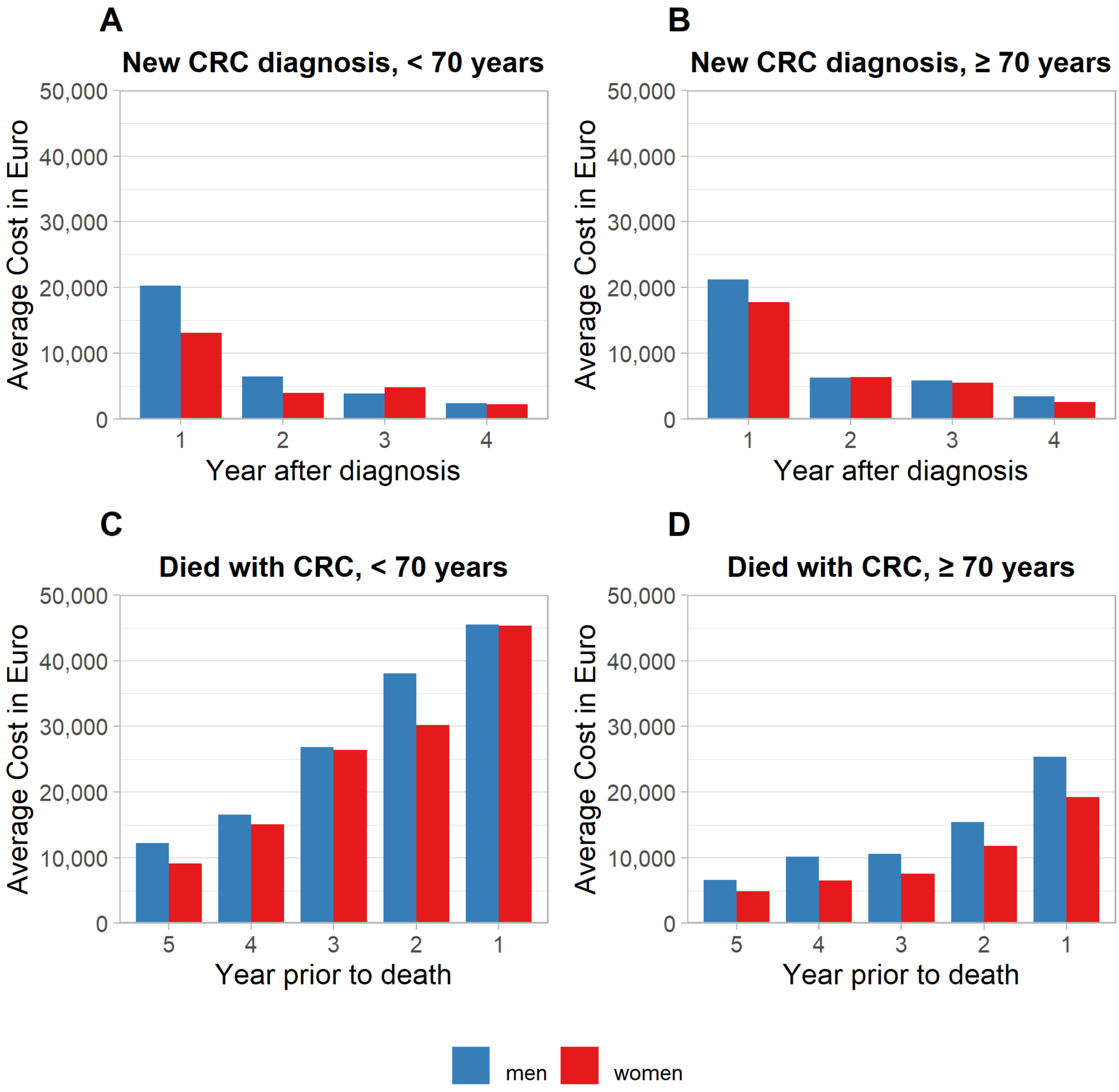

CRC, colorectal cancer

Supplementary Figure S3. Average total healthcare costs in colorectal cancer patients after new diagnosis and prior to death, stratified by sex and age. A+B, newly diagnosed cases. C+D, subjects who died with CRC.

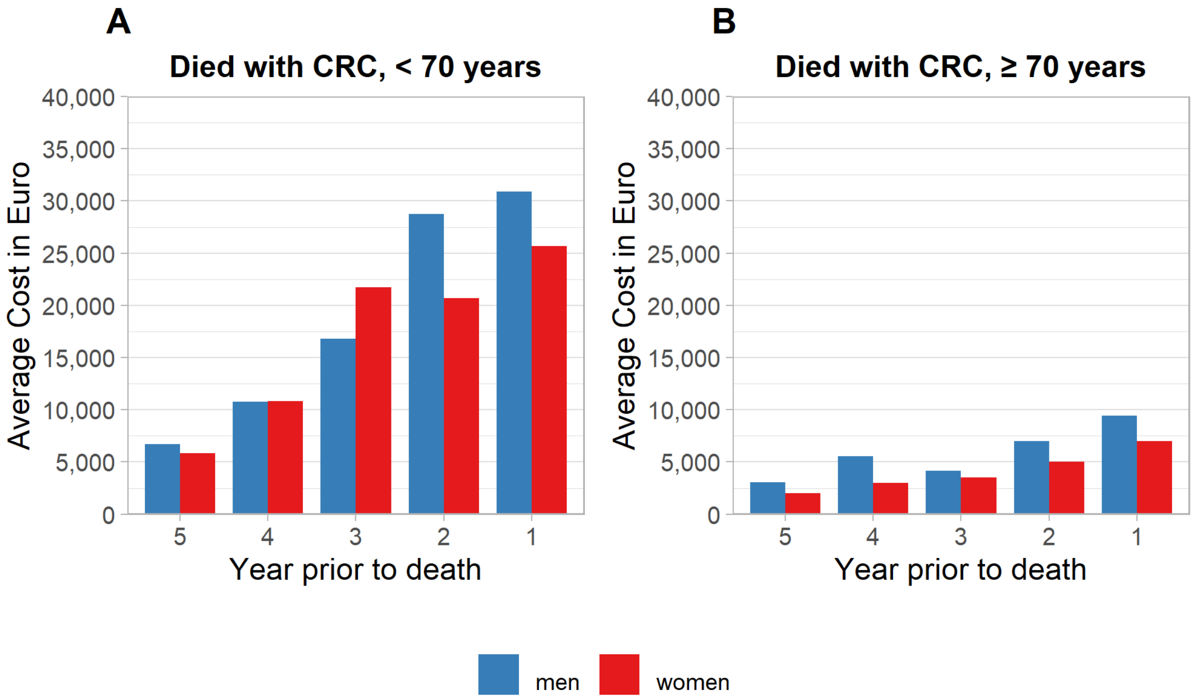

CRC, colorectal cancer

Supplementary Figure S4. Total average treatment costs of colorectal cancer prior to death, stratified by sex and age, excluding individuals with poor prognosis (diagnosed and died within 2012–2016).

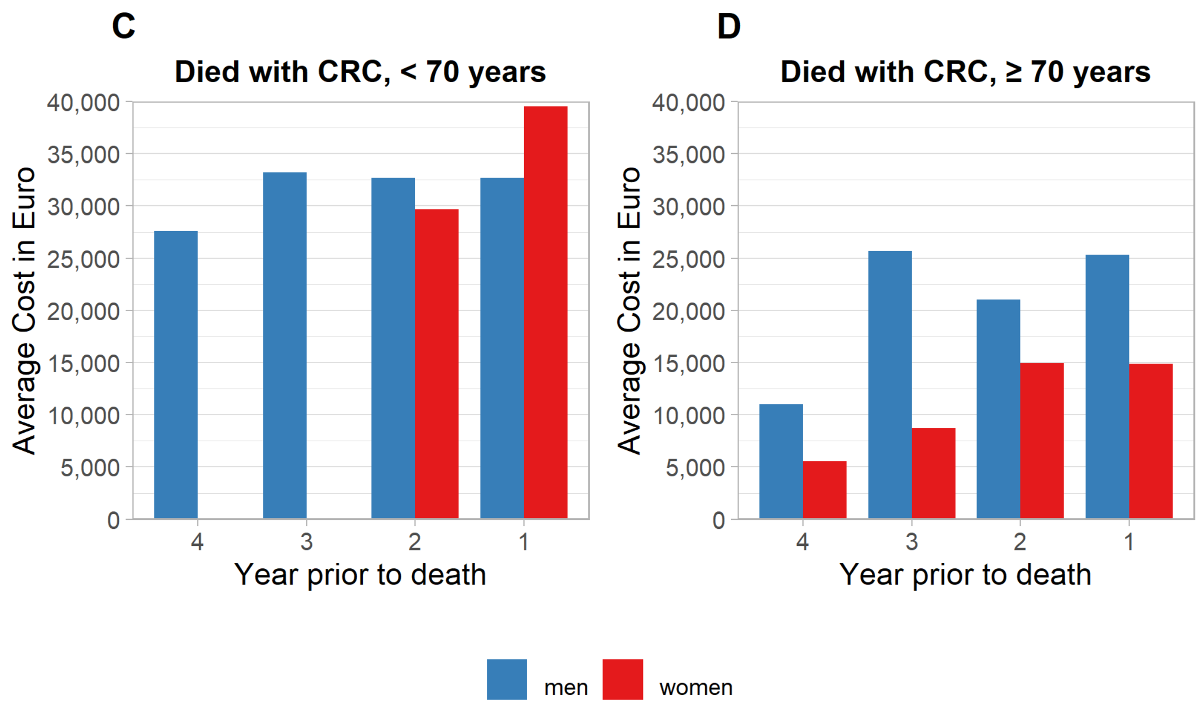

CRC, colorectal cancer
* no data available for women <70 years 3–4 years prior to death

Supplementary Figure S5. Total average treatment costs of colorectal cancer prior to death, stratified by sex and age, only individuals with poor prognosis (diagnosed and died within 2012–2016) *.
